# Coordinated regulation of immune contexture: crosstalk between STAT3 and immune cells during breast cancer progression

**DOI:** 10.1186/s12964-021-00705-2

**Published:** 2021-05-06

**Authors:** Jing Jin, Yi Li, Qijie Zhao, Yue Chen, Shaozhi Fu, JingBo Wu

**Affiliations:** 1 Department of Oncology, The Affiliated Hospital of Southwest Medical University, Luzhou, 646000 Sichuan People’s Republic of China; 2 Department of Nuclear Medicine, The Affiliated Hospital of Southwest Medical University, Luzhou, 646000 Sichuan People’s Republic of China; 3 Nuclear Medicine and Molecular Imaging Key Laboratory of Sichuan Province, Luzhou, 646000 Sichuan People’s Republic of China; 4 Academician (Expert) Workstation of Sichuan Province, Luzhou, 646000 Sichuan People’s Republic of China; 5 Department of Radiologic Technology, Center of Excellence for Molecular Imaging (CEMI), Faculty of Associated Medical Sciences, Chiang Mai University, Chiang Mai, 50200 Thailand; 6 Department of Pathophysiology, College of Basic Medical Science, Southwest Medical University, Luzhou, 646000 Sichuan People’s Republic of China

## Abstract

Recent insights into the molecular and cellular mechanisms underlying cancer development have revealed the tumor microenvironment (TME) immune cells to functionally affect the development and progression of breast cancer. However, insufficient evidence of TME immune modulators limit the clinical application of immunotherapy for advanced and metastatic breast cancers. Intercellular STAT3 activation of immune cells plays a central role in breast cancer TME immunosuppression and distant metastasis. Accumulating evidence suggests that targeting STAT3 and/or in combination with radiotherapy may enhance anti-cancer immune responses and rescue the systemic immunologic microenvironment in breast cancer. Indeed, apart from its oncogenic role in tumor cells, the functions of STAT3 in TME of breast cancer involve multiple types of immunosuppression and is associated with tumor cell metastasis. In this review, we summarize the available information on the functions of STAT3-related immune cells in TME of breast cancer, as well as the specific upstream and downstream targets. Additionally, we provide insights about the potential immunosuppression mechanisms of each type of evaluated immune cells.

**Video abstract**

**Supplementary information:**

The online version contains supplementary material available at 10.1186/s12964-021-00705-2.

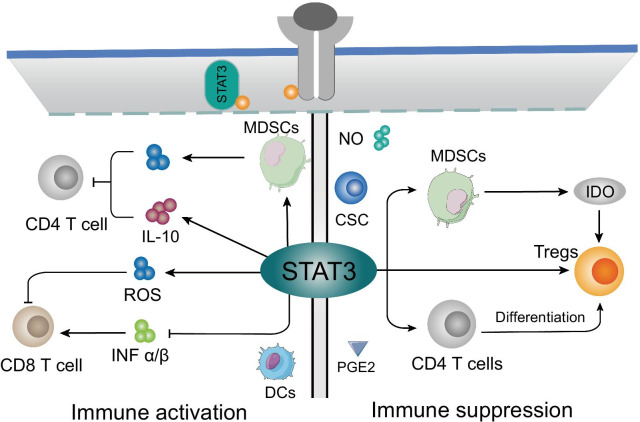

## Introduction

Breast cancer is one of the most prevalent type of gynecological cancer across the globe. As per estimates 2,088,849 new breast cancer cases and 626,679 breast cancer related deaths were reported in 2018 [1]. Owing to its complex pathology, breast cancer is generally diagnosed at advanced stages when it has already spread to different and distant body parts [2]. As a highly malignant tumor, breast cancer exhibits considerable metastatic potential and often leads to treatment failure and death [3]. Breast cancer can be divided into three types: hormone receptor-positive (estrogen receptor (ER) or progesterone receptor (PR)) constitutes 70% of breast cancer cases, ERBB2-positive constitutes 15–20% of breast cancer cases and triple negative breast cancer (ER^−^, PR^−^ and ERBB2^−^) constitutes 15% of breast cancer cases [2]. For management of breast cancer, patients with local disease usually undergo surgery and/or radiation therapy, while the cytotoxic chemotherapy, biologic therapy and endocrine therapy are generally applied to systemic metastasis [4]. Although the breast cancer death rate has decreased by 39% in the last one decade owing to recent advancements in breast cancer diagnostics and therapeutics, almost all patients who are diagnosed with advanced stage and metastatic disease eventually succumb to it [5]. Innovative approaches to reduce frequent relapse of breast cancer and to decrease the death rate is need of the hour. There is concrete evidence in literature that immune system plays an important role in the response of patient reaction to both standard and long-term therapy [6]. The evolving interaction between breast tumor and human immunity was characterized by immunoediting, such as tumor cells death, dendritic cells (DCs) maturation, and effector T cells response [7]. Owing to the heterogeneity of the breast cancer [8], the effect of complex tumor microenvironment (TME) on immunotherapy still lacks sufficient validation.

**Fig. 1:**
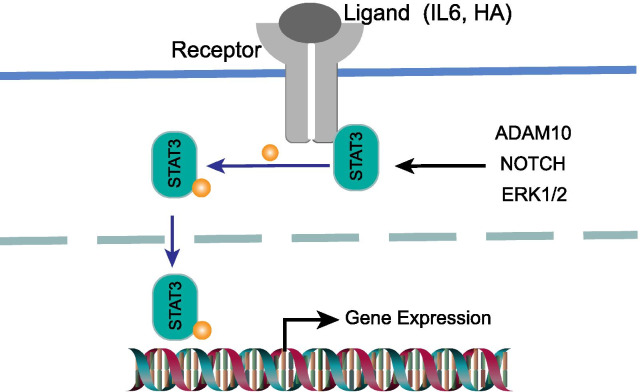
A schematic depiction of constitutive STAT3 activation in immune cells. The phosphorylation of STAT3 is triggered by tumor-derived factors and genetic stress. The activated STAT3 subsequently translocated to the nuclear, where it binds to the DNA at specific site and leads to increased transcription of target genes, thereby contributing to immune cells generation, polarization and immunosuppression properties

STAT3 is the family member of signal transduction and transcriptional activators (STAT) proteins, which regulate the gene expression related to cell survival and immune response associated with tumor progression and malignancy [9–11]. Generally, STAT3 is localised in the cytoplasm of resting cells in an inactive form [12]. Once activated, STAT3 undergoes phosphorylation, homodimerization, nuclear translocation and DNA binding, subsequently driving the tumor proliferation, differentiation, apoptosis, cell transformation, invasion, angiogenesis, and immune evasion [13] (Fig. 1). Inhibitors of STAT3 have been reported to inhibit cell proliferation and promote the apoptosis of lung cancer, gastric cancer, colorectal cancer, leukemia, melanoma, renal cancer and breast cancer, to name a few [14]. Likewise, immunosuppression and tumor promotion are magically integrated into STAT3 cascade [15–17]. Accumulating evidence revealed that STAT3 is an important oncoprotein in an overly complex TME [18–21]. Breast cancers risk, metastasis, recurrence, and response to treatment is affected by multiple non-malignant cell types in TME, such as macrophages, mast cells, B cells, regulatory T cells (Tregs), DCs, and natural killer cells (NKs) [22, 23]. Recently, STAT3 signaling has been shown to pay a role in immune cells and promotes immunosuppressive function in the TME [24]. It is widely accepted that the immunological insult in TME and the activated immunosuppressive molecules in human cancers are essential in modulating the tumor milieu and tumor progression [18, 25]. STAT3 signaling activation is magically converged in both tumor promotion and immunosuppression, such as the crosstalk between tumor cells and immune cells [18]. Meanwhile, the immune system has a key role in the standard treatment response and long-term survival of breast cancer patients [6]. Recent insights indicate that breast cancer outcomes are determined by the type of elicited immune responses [26–28]. Of note, a lot of studies have revealed that STAT3 cascade was associated with breast cancer immune responses [29, 30], which can potentiate signaling in TME immune cells and tumor cells. However, the effects of STAT3 cascade on immune cells in breast cancer tumor immune microenvironment is yet to be elucidated.

In this review, we intend to establish the interaction between STAT3 and immune cells in TME of breast cancer, focusing on the immune cells function and population. Describing above appears to imply an ideal target for breast cancer immunotherapy. STAT3 is not only overactive in different cells of breast cancer milieu, but also simultaneously determines opposite patterns of anti-tumor and pro-tumor immune cells, such as downregulated T cells and upregulated Tregs. The specific inhibition of STAT3 has emerged as a promising strategy to improve the TME, immune surveillance, tumor progression and metastasis of breast cancer. The treatment arms of STAT3 inhibition in combination with radiotherapy exhibits the potential to ameliorate the immunosuppression and favors the systemic immune response.

## STAT3-related immune cells in breast cancer tumor microenvironment

The TME plays a crucial role in tumor progression, treatment response and patient prognosis. Myeloid-derived suppressor cells (MDSCs), DCs, Tumor-associated macrophages (TAMs), tumor-associated neutrophils (TANs), NKs, B cells and T cells are the main immune cells in the TME [31, 32]. As one of many important regulatory factors in TME, STAT3 is a key target that connects the microenvironment with tumor cells [31]. The following is the role of STAT3-related immune cells (MDSCs, macrophages, DCs, and T cells) in the TME of breast cancer (Table 1).

**Table 1:** The role of STAT3 in regulating signaling proteins in immnue cells

Immune cells	Proteins	Relathionship with STAT3	References
MDSCs	IDO	MDSCs-activated STAT3 suppressed the T cell expansion and Th1 polarization via the IDO manner	[37]
	IRF-8	STAT3 downregulted the IRF-8 expression and promoted the MDSCs formation	[44]
	G-CSF	G-CSF mediated the STAT3/IRF-8 axis functions in MDSCs	[45]
	IL-6	IL-6 stimulated STAT3 phosphorylation in MDSCs	[48]
	S100A8/A9	STAT3 stimulated the S100A8/A9-mediated ROS, then suppressed CD4^+^ T cells accumulation	[59]
Macrophages	CD206/Arg-1/PTGS2	STAT3 inhibition suppressed these markers expression	[64, 73]
	HA	HA actived the STAT3 cascade	[72]
	A-FABP	A-FABP stimulated the STAT3 activation by promoting IL-6 production	[67]
	HIF-1α/TGF-β1	STAT3 upregulated HIF-1α/TGF-β1 expression, and influenced angiogenesis, tumor cells proliferation and metastasis	[82]
	PD-L1	STAT3 promoted the PD-L1 secretion on macrophages of tumor milieu	[87]
Dendritic Cells	PKCβII/PRKCB2	STAT3 reduce the PKCβII protein and PRKCB2 expression and suppressed DCs generation	[98]
	HER-2/neu	STAT3 inhibition downregulated the tumor surface HER-2/neu expression	[103]
	IL-10	IL-10-related signaling plays an important role in STAT3-elicited cDCs immunosuppressive response	[105]
	FLT3L	FLT3L promoted DCs proliferation via STAT3-dependent manners	[111]
	Tcf4	STAT3 interacted with Tcf4 promoters and increased the pDCs population	[110]
CD4 + T cells	IL-10	STAT3 increased the IL-10 expression and counteracted CD4 + T cells tumoricidal function	[34]
Tregs	Foxp3	STAT3 directly regulated the expression of Foxp3, and promoted the Tregs generation and immunosuppressive abilities	[138]
	IDO1	STAT3-mediated IDO1 expression increased the Foxp3^+^ Tregs in tumor milieu	[150]
CD8 + T cells	INF-α/β	STAT3-blocking induced INF-α/β production and triggered CD8^+^ T cells responses	[165, 167]
	GAPDH/HK2	STAT3 activation repressed GAPDH/HK2, which were critical glycolic indicators for T cells	[172, 174]
	FGFR4	Genetic instability of FGFR4 enhanced the STAT3 activation and possibly suppressed CD8^+^ T cells infiltration	[137]

## MDSCs

**Fig. 2:**
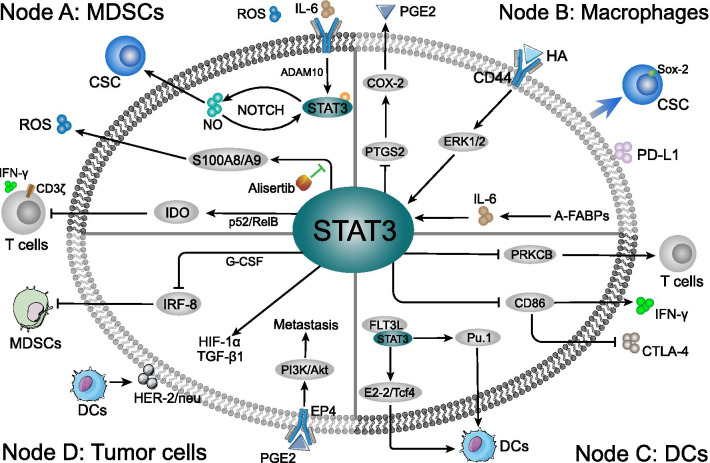
The mechanism of STAT3-related immune cells at in breast cancer TME. Three primer immune cells can be targeted when STAT3 influences the TME of breast cancer. The immune cells population, phenotypes and related gene expression were shaped in tumor milieu. **a** Node A: MDSCs conditioned by STAT3 cascade in TME induced paralysis of T lymphocytes, activity of CSCs, and carcinogenic factors generation. Meanwhile, the release of ROS might enhance the immunosuppression in various routes. **b** Node B: Intracellular STAT3 cascade in the macrophages affects the PGE2 and PD-L1 secretion, and induces the Sox2-positive CSCs in TME. Moreover, the HA and A-FABPs induced STAT3 activation is directly associated with TAMs formation and enables the interaction between tumor cells and macrophages, such as promoting TGF-β1 and HIF-1α generation. **c** Node C: STAT3 cascade suppressed DCs differentiation and deprived the DCs ability to stimulate T cells. Through inhibiting CD86 expression, STAT3 indirectly inhibited the CTLA-4 and promoted IFN-γ expression in TME. Moreover, FLT3L-induced DCs accumulated in immunization site and significantly increased the anti-tumor T cells response and remarkably delayed the tumor growth. The FL3TL/STAT3/Pu.1 cascade promote the differentiation and maturation of DCs, while FL3TL/STAT3 interacts with E2-2/Tcf4 pathway to enhance pDCs-related immune response. **d** Node D: STAT3 cascade in tumor cells inhibits the MDSCs in TME, which was directly mediated by intercellular G-CSF/IRF-8 function. The co-culture between tumor cells and DCs stimulated STAT3-related HER-2/neu, TGF-β1 and HIF-1α generation. Moreover, the macrophages related PEG2 in TME might stimulate PI3K/Akt pathway via the tumor surface EP4 receptor recognition, which was closely connected to breast cancer cells metastasis

As a heterogeneous population of myeloid progenitor cells in TME, myeloid-derived suppressor cells (MDSCs) are associated with inflammation, tumor progression and metastasis [33, 34]. By suppressing CD4^+^ T cells, CD8^+^ T cells and NK cells, recruited MDSCs can inhibit innate and adaptive immune response, leading to the eliminative dysfunction of immune system and suppression of immune surveillance [33, 35]. While markers for the heterogeneous MDSCs have not been defined well because different tumor types have different markers for MDSCs [36]. In breast cancer, phosphorylated-STAT3 directly induced indoleamine 2,3-dioxygenase (IDO) expression in MDSCs by binding to the promotor of IDO which is involved in immunosuppressive effects between breast cancer-derived MDSCs on T cells [37] (Fig. 2a). IDO, a rate-limiting enzyme in tryptophan catabolism, is highly expressed in MDSCs isolated from fresh breast cancer tissues and is associated with tumor-induced immunosuppression by suppressing T cell function [38, 39]. Moreover, MDSCs have been reported to activate the STAT3 mediated inhibition of T cell expansion and Th1 polarization via the IDO manner in breast cancer [39]. Meanwhile, STAT3 cascade blocking has been shown to significantly decrease the IDO expression in MDSCs, tumor development, and metastasis [40]. Noteworthy, intranuclear p52 and RelB (p52/RelB complex) has also been found to be dramatically decreased after STAT3 blocking, which is a dimer involved in NF-κB pathway activation and specific immunological processes [41], implicating that noncanonical NF-κB pathway participated in the STAT3-induced IDO expression and TME immunosuppression [37, 42]. Through the negative association between IDO and T cells CD3ζ-chain/IFN-γ expression, activated IDO from the MDSCs directly abolished the T cells immunity in TME [43]. Furthermore, STAT3 has also been shown to be negatively associated with the downstream interferon regulatory factor–8 (IRF-8) expression via the promoter engagement. This eventually promotes the MDSCs phenotype, which is directly mediated by G-CSF in breast cancer [44, 45]. Downregulation of IRF-8 has been found to facilitate the development and accumulation of MDSCs [44, 46] (Fig. 2d). Therefore, STAT3-related MDSCs generation is considered as a major obstacle to anti-tumor immunotherapy.

For the upstream, reactive oxygen species (ROS) and IL-6 triggered STAT3 activation regulates MDSCs expansion in the breast cancer, which are both typical characteristics of MDSCs [40, 43, 47]. The IL-6 level in TME has been shown to be positively correlated with infiltration of MDSCs in situ and contributed to worse clinical outcomes [48, 49]. Among which, ADAM proteases (ADAM10)-induced soluble IL-6 was particularly involved in IL-6 trans-signaling and accompanied with the enhanced STAT3 phosphorylation in MDSCs (Fig. 2a). Additionally, infiltrated MDSCs might facilitate the shedding of IL-6 receptor, eventually promoting invasion and metastasis of breast cancer cells via IL-6 trans-signaling [50]. With the IL6-dependent STAT3 stimulation, MDSCs-derived nitric oxide (NO)NO/NOTCH signaling can promote and maintain the persistent phosphorylation of STAT3 [51]. Furthermore, cancer stem cells (CSCs) are associated with tumor progression and treatment resistance [52]. Through STAT3 and NOTCH signaling, MDSCs induced human breast CSCs in TME and exhibited poor survival rate [51]. Among which, IL-6 and MDSCs-derived NO collaboratively stimulated STAT3 and NOTCH signaling. In addition to IL-6, IL-17 appeared to positively regulate the differentiation of MDSCs into macrophages and mDCs, as well as activation of STAT3 [53]. Data also indicated that the levels of IL-17 were considerably downregulated in patients with breast cancer as compared to the healthy blood donors [53].

Recently, Yin et al. reported that the inhibitor of Aurora-A kinase (Alisertib) plays a remarkable role in regulating the immunosuppressive functions of STAT3 and MDSCs in the TME of breast cancer [54]. Aurora-A is a conserved serine/threonine kinase and is associated with poor prognosis and drug resistance [55, 56]. Of note, Alisertib has been used in many preclinical studies and clinical trials, including a recent five-arm phase II study against advanced breast cancer and small cell lung cancer [57]. Alisertib can significantly decrease the MDSCs and enrich T lymphocytes in the TME of breast tumor and peripheral organs, which were essential for the breast cancer regression. Indeed, the administration of alisertib ameliorated immunosuppressive function of MDSCs by inhibiting STAT3-mediated ROS generation in breast cancer (Fig. 2a) [54]. Activated-STAT3 (Tyr705) and ROS levels have been shown to significantly downregulated in MDSCs from alisertib-treated mice. T-cell suppressive activity of MDSCs has been found to be positively associated with ROS generation in a STAT3-dependent manner [58]. Moreover, S100A8/A9 (two modulators of the ROS generation in MDSCs) expression was considerably decreased after alisertib treatment [59, 60], which both have been demonstrated to be regulated by STAT3 [61]. Therefore, constitutive activation of STAT3 regulates MDSCs and anti-cancer T lymphocytes population in breast cancer TME.

## Macrophages

Macrophages are essential for host defense and can be divided into two distinct forms, M1-macrophages and M2-macrophages. While the M1-macrophages produce IL-12 to promote Th1 response, and the M2-macrophages sustain Th2-associated effector functions and secrete tumor supportive factors [62, 63]. TAMs are closely similar to M2-polarized macrophages and promote immune evasion of breast tumor cells [64, 65]. Clinical and experimental data demonstrated that a high density of TAMs is associated with both poor prognosis and metastasis in breast cancer patients [66–68]. In TME of breast cancer, ERK/STAT3 cascade has emerged as a pivotal regulator to stimulate macrophage M2-like polarization and promote tumor progression and metastasis [64]. Accordingly, STAT3 inhibitor in combination with ERK inhibitor has been found to significantly suppress the M2 macrophage polarization and expression of markers including CD206 and Arg-1. Likewise, the expression of hyaluronan (HA) in TME of breast cancer patients is positively correlated with the amount of M2 macrophages [69] (Fig. 2b). HA is the most important component of extracellular matrix (ECM) and is associated with poor prognosis of breast cancer [70]. With the recognition by HA major receptor CD44 on macrophages [71], TME-derived HA activated the STAT3 cascade and formation of TAMs [72]. In addition, STAT3-inhibitor S3I-201 [72], simultaneously suppressed the STAT3 phosphorylation and macrophages transformation in breast cancer TME as compared to control. Meanwhile, STAT3 blocking in macrophages is positively associated with PTGS2 expression, which triggers the cyclooxygenase-2 (COX-2) to drive PGE2 generation [73]. Intriguingly, increased pro-tumorigenic factor COX-2 in TME might contribute to the attenuated therapeutic responses of breast cancer to ruxolitinib and promote tumor progresses [73, 74]. The PGE2 has been reported to active AKT via PI3K signaling pathway, which is associated with tumor cells proliferation and survival [75, 76]. Consistently, COX-2-mediated PEG2 expression has been shown to be intricately connected to lymph angiogenesis and lymphatic metastasis via PI3K/Akt-dependent receptor EP4 recognition in breast cancer cells [77, 78] (Fig. 2d). Thus, the pros or cons of STAT3 cascade target therapy need to be further illustrated.

Recently, Hao et al. reported the intracellular adipocyte/macrophage fatty acid binding protein (A-FABP) expression to be negatively associated with breast cancer survival via facilitation of the STAT3 cascade in TAM [67] (Fig. 2b). FABP does not only regulate the inflammatory and cellular metabolic pathways, but also affects the macrophages function and phenotype [79–81]. A-FABP has been shown to exhibit the ability to promote IL-6 production through NFκB/miR29b pathway in macrophages, which eventually boosted the phosphorylation of STAT3 [67]. In contrary, A-FABP knockdown or anti-IL-6 significantly decreased the STAT3 phosphorylation level in macrophages, subsequently inhibiting the tumor colony formation and metastasis. Moreover, IL-6 activated the STAT3 was specifically enriched in co-culture system between macrophages and breast cancer MCF-7 cells as compared to control, which also showed significant upregulation of transforming growth factor β (TGF-β1) and hypoxia-inducible factor-1α (HIF-1α) mRNA levels [82] (Fig. 2d). Noteworthy, HIF-1α, as a STAT3 downstream target, has been proved to be associated with angiogenesis [83, 84]. STAT3 positively has been shown to positively regulate the expression of TGF-β1 in breast cancer [85], thereby promoting cancer cell proliferation and metastasis [86]. Another co-culture system between macrophages and 4T1 cells revealed that STAT3 was not only activated in macrophages, but also promoted the PD-L1 secretion on macrophages during interaction with breast cancer cells (Fig. 2b) [87]. High PD-L1 expression macrophages have been reported to directly promote apoptosis and inhibit the proliferation via suppressing of activation of autologous T cells [88]. Of note, the interaction between PD-L1 and macrophages surface PD-1 induced an alternative M2-like polarization, resulting in crippling effects on tumor immunity [89]. Furthermore, the crosstalk between TAMs and breast cancer cells showed an ability to induce CSCs phenotype formation with upregulated Sox-2 expression via activation of STAT3 cascade [90]. Accordingly, Sox-2 expression was directly inhibited by STAT3 inhibitor 2-cyano-3,12 dioxooleana-1,9 dien-28-imidazolide (CDDO-Im) treatment. Increased CSCs have been shown to boost the metastasis, chemotherapy resistance, and tumorigenicity in vivo [90, 91]. Although targeting TAMs is a promising clinical tumor immunotherapy strategy, it is difficult to achieve in practice due to the heterogeneous and dynamic nature of macrophages in TME [63, 92, 93]. Against this backdrop, modulating the intercellular STAT3 cascade of macrophages may provide an opportunity to improve therapeutic efficacy of the breast cancer immunotherapy.

## Dendritic cells

As primary antigen-presenting cells (APCs), conventional dendritic cells (cDCs) play a main role in adaptive immune response initiation and regulation [94, 95]. The cDCs consist of type 1 (cDC1) and type 2 (cDC2) subsets [96]. In immune system, DCs suppress cancer growth and spread, even when the disease has advanced [97]. The presence of DCs in TME is positively correlated with patient survival. It is now thought that tumor-derived factors (TDFs) IL-6, VEGF, and G-CSF secreted by tumor itself in TME of breast cancer have the ability to stimulate STAT3 cascade in myeloid cells differentiation [34, 98]. As a result, STAT3 activation impaired the generation of DCs as well as DCs function. Mechanically, in both advanced breast cancer patients and breast tumor-bearing mice, STAT3 directly decreased the PKCβII protein and PRKCB2 expression by binding to negative regulatory elements of the PRKCB promoter [98], eventually leading to the suppression of DCs formation and this subtle change can delay the T cells activity, namely CD8^+^ CTLs (Fig. 2c) [99, 100]. PKCβII has been deemed as splice variant of the PRKCB gene [101], which drives the differentiation of myeloid progenitor cells to DCs [99, 102]. As a negative feedback loop, PKCβII activity inhibited the ability of TDFs to activate STAT3 cascade, via reduction of abundance of the cell surface receptors recognition to TDFs [98]. Knock down of STAT3 in TME of breast tumor results in the downregulation of the surface expression of on tumor cell via the DCs activation [103], and HER-2/neu is a proto-oncogene linked to breast cancer progression and metastasis [103, 104]. Recently, STAT3-deficient cDCs (STAT3^−^ cDCs) was reported to inhibit the breast cancer growth and poor survival as a cell-based vaccine. STAT3^−^ cDCs injection was positively associated with antigen-specific T cells accumulation in breast TME and tumor-related lymph nodes compared to blank control [105]. In addition, upregulated surface CD86 expression was observed rather than CD80 or MHC II due to STAT3 abolishment. DCs-generated co-stimulatory activation (CD86) inhibited the CTLA-4 secretion in breast cancer lymph nodes, promoted intercellular IFN-γ expression, and recognized the CD28 receptor in T cells, therefore promoting T cell accumulation and activity [106–108] (Fig. 2c). Furthermore, with the specific receptor IL10RB interaction, IL-10-related signaling plays an important role in STAT3-elicited cDCs immunosuppressive responses of breast TME [105]. Ablating the IL-10/STAT3 cascade dramatically improved the effector T cells amounts. Consistently, increased amount of T cells in TME was linked to efficacy of immunotherapy in breast cancer [109]. The inhibition of STAT3 in migratory cDCs might be a novel immunotherapy strategy for management of metastatic and advanced breast cancer [105].

Intriguingly, another important DCs growth regulator, FMS-related tyrosine kinase 3 ligand (FLT3L) showed the ability to promote DCs proliferation via STAT3-dependent manner [110, 111]. STAT3 activation acts as a checkpoint of FLT3L-regulated DC diversification (Fig. 2c). Moreover, in TME of breast cancer, FLT3L-induced DCs accumulated in immunization site and significantly increased the anti-tumor T cells response and remarkably delayed the tumor growth [112, 113]. While the expression of FLT3L is prevalent in lymphoid (60%–70%) and myeloid (50%–65%) progenitors, absence of STAT3 restricted myeloid differentiated into DCs progeny than T and B lymphocytes [111]. Therefore, STAT3 is a non-redundant regulator of FLT3L-mediated DCs differentiation. One hypothesis is that STAT3-actived Pu.1 expression in lymphocyte and myeloid cells differentiation [114, 115], and high level of FLT3L initiated DCs differentiation via the STAT3/Pu.1 cascade [116]. Nonetheless, the high expression of IL-32 in breast cancer might suppress the STAT3 binding to the Pu.1 promoter, thereby leading to the abrogated maturation of DCs and tumor progression [114, 117].

Furthermore, in plasmacytoid DCs (pDCs), FLT3L used STAT3 to selectively induce the E protein E2-2/Tcf4 interaction, which is essential for the pDC population (Fig. 2c), but not for the other DC lineages in TME [110, 118]. Among which, STAT3 physical interacted with Tcf4 promoters suggesting a direct mechanism of gene induction [110]. Although it is generally assumed that binding of β-catenin to members of the TCF family is cancer-promoting, recent studies have shown that Tcf4 functions instead as a repressor that restricts the breast cancer cell growth [119]. In addition, activation of pDCs contributed to higher killing efficacy of effector lymphocyte in TME of breast cancer model, including FLT3L-induced pDCs [120, 121]. In yet another study, simultaneous inhibition of STAT3 and Tcf4 signaling pathways was reported to suppress the breast cancer cells metastasis both in vitro and in vivo [122]. Despite insufficient evidence, FLT3L/STAT3 cascade directly conducted pDC and anti-tumor immunity via Tcf4 in breast cancer. As such Tcf4 might be used act as a new prognostic biomarker and valuable molecular target for breast cancer immunotherapy.

## STAT3-relevant T cells immunosuppression

**Fig. 3:**
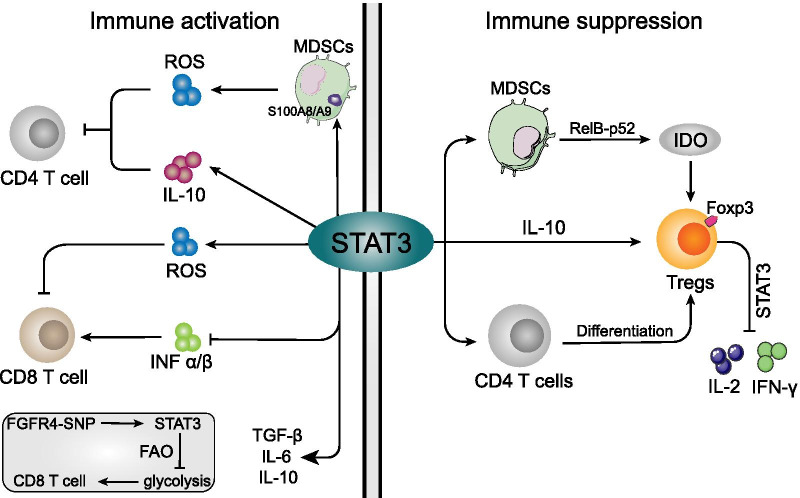
The roles of STAT3 in different immune cells immunosuppression. **a**: For immune activation cells (Left panel), STAT3 directly regulate MDSCs to product IL-10 and ROS in TME, both of which indirectly cause CD4^+^ T cells to lose their ability to inhibit tumors. The STAT3-induced ROS impaired the CD8^+^ T cells, while STAT3 also indirectly suppress the CD8^+^ T cells through inhibiting the INF-α/β generation in breast cancer TME. As a nonredundant regulator of CD8^+^ T cells, activated-STAT3 was also involved in intercellular glycolysis via promoting the FAO expression. **b** For immunosuppressive cells, however (Right panel), STAT3 not merely promote the Tregs through triggering IDO (RelB-p52 binding) and IL-10 generation from MDSCs, but also was directly involved in Tregs specific marker Foxp3 expression and immunosuppressive effect. The activated-STAT3 promoted the naive peripheral CD4 + T cells conversion into Tregs, simultaneously inhibiting the IL-2 and IFN-γ production from converted lymphocytes

STAT3 is constitutively activated in all subtypes of breast cancers and particularly plays an essential role in their immunosuppression [123]. Activated-STAT3 in tumor cells not only dampens the generation of immunostimulatory mediator, but also boosts the release of the immunosuppressive factors leading to the stimulation of crosstalk between tumor cells and T cells in TME [124] (Fig. 3). Herein, we discuss the STAT3 related T cells immunosuppression in breast cancer as follows.

### CD4^+^ T cells

As previous study has reported that blocking of STAT3 in breast cancer cells induced an antitumor immune response involving CD4^+^ T cells (Fig. 3a), which may ameliorate TME via secretion of cytokines, such as TNF-α, IFN-γ, IL-6, and IL-5, as well as chemokines CCL5 and CXCL10 [125]. Importantly, TNF-α and IFN-γ synergistically induced signaling in CD4^+^ T cells that prevents immune evasion, tumor cell proliferation, and multistage carcinogenesis [126, 127]. Moreover, tumor-infiltrating NKs and MDSCs underwent transcriptional reprogramming and enhanced the IL-10 production via STAT3 in breast cancer [58, 128]. As a result, increased IL-10 in TME directly suppressed the generation of CD4^+^ T cells, thereby impairing tumor immunity. Moreover, IL-10 has pro-tumor activity in TME, and IL-10 facilitated Tregs and M2-like macrophages development [129], as well as counteracting CD4^+^ T cells, cytotoxic CD8^+^ T cells and NKs tumoricidal function [34]. Therefore, IL-10 in TME of breast cancer induces immunosuppression and assists the evasion from tumor immune surveillance, promoting tumor cell development and metastasis [130]. Furthermore, much of the suppressive activity of T cells is due to the ROS production, including CD4^+^ T cells [131–133]. The S100A8/A9-mediated ROS suppression was reported to improve the CD4^+^ T cell accumulation in TME of breast cancer, which has been known to be regulated in STAT3-dependent mechanism in MDSCs [54, 58, 59, 61].

### Tregs

Tregs is a subset of CD4^+^ T cells which sustain the immunosuppressive environment in human cancers. Generally, Tregs trigger immunosuppression by initiating competition for microenvironment factors, thereby leading to cytokine deprivation-induced apoptosis in the target effector T cells [134]. Olkhanud et al. showed that Tregs to be necessary for breast cancer cell metastasis to lungs, which was accompanied with chemokine receptor-mediated chemotaxis and killing function of NKs [135]. In breast cancer animal model with tumor metastasis, activated STAT3 and higher tumor-specific Tregs population showed co-existence and contributed to immunosuppression [136] (Fig. 3b). Moreover, increased intercellular STAT3 in Tregs resulted in enhanced proliferation and suppressive functions of Tregs, further supporting the STAT3-associated Tregs immunosuppression in TME of breast cancer [137].

Furthermore, the expression of Forkhead box P3 (Foxp3), a fundamental mediator of Tregs, is directly regulated by intercellular STAT3 cascade [138]. Activated Foxp3 can bind to a proximal site of the il2 promoter in vivo, leading to the upregulation of several Treg-associated markers expression [135]. The molecular events driving Foxp3 expression required T-cell receptor (TCR)/CD28 to stimulate STAT3 transcription, which in turn promoted the naive peripheral CD4^+^ T cells conversion into Tregs and acquisition of Tregs suppressive function. Moreover, it also caused, inhibition of IL-2 and IFN-γ production from converted lymphocytes [139, 140]. The positive function of STAT3 is that it can bind to the Foxp3 locus (Exon 2 β sheet region) and promote gene expression, thereby providing an important mechanism by which STAT3 can promote Tregs [141–143]. Meanwhile, Tregs lacking STAT3 are devoid of expansion, differentiation and immunosuppressive abilities. Moreover, Treg number has been found to be decreased in the TME of STAT3-deficient mice [139]. Specifically, elevated Foxp3 gene expression and accumulated Foxp3^+^ Tregs amount were detected in close proximity to lung metastases of breast cancer, as well as higher STAT3 activities [144]. STAT3 cascade may elicit immunosuppressive TME by recruiting Foxp3^+^ Tregs to the metastatic milieu. Concordantly, some studies have revealed that blocking of STAT3 in breast cancer animals significantly decreased the Tregs proportion in the TME especially the Foxp3^+^ Tregs [30, 125, 145]. Additionally, STAT3-blocking suppressed the pro-inflammatory and the anti-inflammatory Tregs, thereby adding to T cell cytotoxicity which is conducive for the anti-tumor effects [30].

Previously, Kyung et al. has reported that target TME Tregs regulator indoleamine-2,3-dioxegenase 1 (IDO1) achieved great success in various tumor types (11% achieved partial response or complete response), including breast cancer [146]. IDO was firstly found in 1950s and inhibits the T cell immunity by inducing differentiation and maturation of Treg cells, which is a poor prognosis factor for breast cancer [147–149]. Notably, MDSCs-produced IDO1 favors Foxp3 + Tregs and tumor-induced immunosuppression, which in turn leads to advanced breast cancer clinical stage [150, 151]. Furthermore, STAT3-mediated IDO1 expression was found to be upregulated in breast cancer cell-induced MDSCs, which suppressed effector T cells and hyperactivated the infiltration of the Foxp3^+^ Tregs in TME [37, 151, 152]. In contrary, STAT3 blocking downregulated the IDO1 proteins in MDSCs and stimulated T cell proliferation [37, 152]. Yu et al. showed that STAT3 activation induced IDO through NF-κB activation rather than by its directly binding function, in which, NF-κB subunits (RelB-p52 dimers) directly bind to the IDO promoter thereby triggering IDO expression [40]. Although STAT3 has been reported to be strongly associated with MDSCs and IDO1 in interacting with Foxp3^+^ Tregs, the STAT3-dependent regulation of IDO1 expression still remains unclear. Therefore, elucidating concrete molecular mechanisms modulating the STAT3/Tregs in breast cancer derived TME may prove essential in the development of novel immunotherapy strategies.

### CD8^+^ T cells

T cells play a central role in human adaptive immune response to cancer, especially the function of CD8^+^ T-cell to kill malignant cells [25]. Tumor-infiltrating CD8^+^ T cells in TME are related to distinct clinical outcomes and survival in breast cancer [153]. STAT3 has been demonstrated to be an important checkpoint that blocks anti-tumor immune responses in a variety of immune cells, especially CD8^+^ T cells [154–157] (Fig. 3a). Recently, Li et al. reported that inhibition of STAT3 activity in breast cancer metastasis model simultaneously impaired matrix metalloproteinase (MMP)-2/9 and ROS generation and increased the CD8^+^ T cells in the TME [158]. Noteworthy, MMP-2 and -9 expression has been reported to enhance CD8^+^ T cells response in liver [159], and MMP-9 is known as a mediator of T cells migration [160, 161]. While, decreased ROS ameliorates the TME-mediated CD8^+^ T cells suppression [54, 162]. Therefore, STAT3-mediated ROS accretion might play a pivotal role in CD8^+^ T cell responses of breast cancer than MMPs. Apart from this, STAT3 inhibition can regulate the production of various immunomodulator factors in TME of breast cancer, such as upregulation of INFs, GM-CSF and IL-2, downregulation of TGF-b, IL-6, and IL-10 proteins [163]. These factors regulate inflammatory and antitumor functions of immune cells, including CD8^+^ T cells [164]. Similarly, it has been reported that blocking of STAT-3 in mice caused significantly higher activation of CD8^+^ T cells in TME as compared to the control [163]. Consistently, STAT3 blocking in 4T1 syngeneic mouse markedly suppressed the tumor by triggering CD8^+^ T cells priming to eliminate tumor cells [165], which may be manipulated by STAT3-blocking triggered INF-α/β production [166, 167]. The CD8^+^ T cells in 4T1 mouse model were also found to decrease the tumor volume, inhibit lung metastasis and prolong the overall survival (OS) [168]. Reports have also revealed that INF-α/β can stimulate the CD8^+^ T cells [169]. Hence, it is possible that high levels of STAT3-mediated IFN-α/β in the TME favors the function of CD8^+^ T cells.

Of note, it has been reported that increased 705-tyrosine phosphorylated STAT3 (STAT3-pY705) level was detected in CD8^+^ T cells of breast tumor tissues [170]. The stimulation of the human breast cancer CD8^+^ T cells also requires intercellular STAT3 regulation. For example, in the TME of breast cancer, STAT3 activation promotes the expression of fatty acid oxidation (FAO) in CD8^+^ T cells, which subsequently inhibits the cellular glycolysis and other functions [170]. Wang et al. also showed that FAO pathway in breast cancer stem cells is regulated by JAK/STAT3 signaling pathway [171]. Moreover, inhibition of CD8^+^ TEFF cells via STAT3 decreased breast tumor burdens and lung metastasis incidence in the Mouse Mammary Tumor Virus-Polyoma Virus Middle T antigen (MMTV-PyMT) transgenic mice. Meanwhile, it has been found that obesity-triggered breast tumor reduced the tumor-infiltrating CD8^+^ TEFF cells and promoted its progression [170]. In addition, STAT3 activation repressed the glyceraldehyde 3-phosphate dehydrogenase (GAPDH) and hexokinase II (HK2), both of which are essential glycolic indicators for T cells [172–174]. Conversely, blocking the FAO has been shown to cause significant promotion of CD8^+^ T cells accumulation and breast cancer regression. On the other hand, genetic instability of SNP allele rs351855-A in fibroblast growth factor receptor 4 (FGFR4) enhances STAT3 cascade and shapes TME in multiple cancer types, attributed to STAT3-pY705 elevation [137]. The suppressed levels of infiltrated CD8^+^ T cells in TME were observed in rs351855-A transgenic breast cancer mouse model, as a systemic trait, which determined the immune evasion in the TME and accelerated the tumor progression [137]. Thus, host-specific genetic variants might dictate immunosuppressive crosstalk between tumor cells and CD8^+^ T cells through STAT3 pleiotropic functions in T cells. Similarly, targeting of the STAT3 upstream, downregulating the intercellular pSTAT3-Y705 activity and upregulating glycolysis by leptin or PD-1 intervention markedly ameliorated the CD8^+^ T effector cells function in TME and prevented the development of breast cancer [170]. Metabolic reprogramming to regulate the function of T cells and upregulate the glycolysis in CD8^+^ T effector cells can promote their anti-tumor activity and IFN-γ production [175, 176]. Collectively, these findings suggest that targeting STAT3 may lead to a potent anti-tumor T cells immune response in breast cancer with pleiotropic functions.

## Targeting STAT3 for breast cancer immunotherapy

The gene-therapy strategies were designed to inhibit the STAT3 signaling and improve the TME in the breast cancer model have proved the potential of STAT3 as a valid target for immunotherapy [177–179]. Inhibition of STAT3 activity by ruxolitinib can remarkably inhibit the breast cancer invasion in vivo [180]. Blocking of STAT3 in breast cancer not only suppressed the tumor progression, but also conferred sensitivity to chemotherapeutic drugs [181]. Apart from these advantages, STAT3 inhibition was recently proposed to improve innate and adaptive anti-tumor immunity and immune surveillance [182]. STAT3 inhibition in tumor cells increases the expression of cytokines and chemokines that ameliorate the TME, including DCs and tumor-specific T cells response [182]. Moreover, targeting STAT3 in human breast cancer cells was reported to suppress the tumor progression by regulating the expression of crucial proteins in tumor milieu, such as survivin, chemokines (CCL5 and CXCL10) and proinflammatory cytokines (IL-6, IL-5, TNF-a, and IFN-γ) [183–185]. In breast cancer, the STAT3 inhibitors presented a positive feedback in tumor intervention. The direct antitumor effect of STAT3 inhibitors alone has been established in several pre- clinical breast cancer studies (Table 2). As previously reported, preclinical studies of small-molecule STAT3 inhibitor S3I-201 and C188 significantly retarded the breast cancer cell growth in xenografts [186, 187]. Stattic, a nonpeptidic small molecule has been shown to selectively inhibit the phosphorylation, dimerization, and nuclear translocation of STAT3, which consequently promotes the STAT3-dependent breast cancer cell death [188]. Moreover, another low-molecular compound STA-21 has also been identified as a selective STAT3 inhibitor that influences the STAT3 dimerization and DNA binding ability in breast cancer [189]. In addition, an antidiarrheal agent (nifuroxazide) was also identified as a potent inhibitor of STAT3, which markedly inhibited the phosphorylated-Stat3Tyr705 in breast cancer, and induced cancer cell apoptosis in a dose-dependent manner [190]. Additionally, the nifuroxazide also been found to exhibit the potential to inhibit the breast cancer metastasis without detectable toxicity, and the decreased MDSCs in lungs as ascertained in mouse model [190]. Recently, different types of novel inhibitors have been proposed. For instance, Eucannabinolide (Euc) suppressed the STAT3 activation and DNA binding capacity, eventually leading to the inhibition of breast cancer cell viability, proliferation and metastasis [191]. Similarly, 10,11-dehydrocurvularin (DCV), a natural-product macrolide derived from marine fungus, has been shown to selectively suppress the STAT3, to consequently inhibit the proliferation, migration and invasion of breast cancer cells (MDA-MB-231 and MDA-MB-468), via induction of apoptosis [192].

**Table 2:** Application of STAT3 inhibitors in breast cancer treatment

Cell Lines	In Vitro or In Vivo	Inhibitors	Radiation	Effects	References
BT474R/NCI-N87R	In vitro	S3I-201	NO	Inhibits STAT3 activation and sensitizes resistant cells to trastuzumab treatment	[186]
SUM159/BT549	In vitro/vivo	C188	NO	Inhibits STAT3 activation (SH2 domain) and ameliorates chemoresistant, like in combining with docetaxel	[187]
MDA-MB-231/ 435S	In vitro/vivo	Stattic	NO	Inhibits STAT3 activation, dimerization, and nuclear translocation	[188]
MDA-MB-468/ 435/MCF7	In vitro	STA-21	NO	Selectively inhibits STAT3 DNA binding capacity and dimerization (did not affect the STAT3 upstream regulators)	[189]
4T1/MCF-7/MDA-MB-231	In vitro/vivo	Nifuroxazide	NO	Inhibits STAT3 activation, MMP-2/9 expression; decreases MDSCs in lung cancer	[190]
MDA-MB-468/231	In vitro/vivo	Euc	NO	Inhibits STAT3 activation and nuclear translocation	[191]
MDA-MB-231/ 468	In vitro/vivo	DCV	NO	Selectively inhibits STAT3 activation, but does not affect the upstream JAK1/2 and silent STAT3	[192]
MCF-10A/7;					
MDA-MB-231/468/T47D,	In vitro/vivo	ODZ10117	NO	Inhibits STAT3 activation (SH2 domain), regardless of other STAT family proteins and upstream regulators	[205]
Breast cancer sentinel lymphocyte	In vitro	AG490	NO	Inhibits the CpG-induced STAT3 activation; promotes DCs maturation and Th1 cells accumulation	[198]
4T1/MDA-MB-231/MCF-7	In vitro/vivo	Pectolinarigenin	NO	Inhibits STAT3 activation, MMP-2/9 expression; improves CD8^+^ T cells recruitation	[158]
4T1	in vitro/vivo	Alisertib	NO	Inhibits STAT3-mediated ROS generation in breast cancer; ameliorates MDSCs immunosuppressive function	[54]
TM40D-MB/TUBO	In vitro/vivo	Pyrimethamine (PYR)	NO	Blockes STAT3 activity; decreases the frequencies of Foxp3 + Tregs and promotes the CD8^+^ T cell	[30]
MDA-MB-231/468	In vitro/vivo	Niclosamide	YES	inhibits STAT3 and Bcl-2, and increases ROS generation in vitro and in vivo; it is identified as a radiosensitizer	[222]
SKBR3	In vitro/vivo	S3I-201	YES	Inhibits STAT3 activation (radiation-related) and increases radiation-induced cell death	[223]

Inactivation of STAT3 contributed to breast cancer immunogenic phenotype, which involved the participation of CD4^+^ T cells and NKs, and decreased Tregs in the TME [125]. In addition, along with the TME immune response, visible inhibition of the lung metastasis was observed in inhibitor group (STAT3-pY705) as compared to the blank control. Consistently, Mara et al. indicated that ablating STAT3 in both breast cancer and melanoma was associated with the activation of CD4^+^ T cells and NKs [145]. Meanwhile, combination of the STAT3 inhibitor and anti-PD-1 antibody synergistically improved T cells activation, CCL5 and IFN-γ releasing in the TME as compared toanti-PD-1 alone, and decreased tumor-infiltrating Tregs, therefore inhibiting the breast cancer progression and metastasis [145]. STAT3-blocking induced CCL5 is important for T cell proliferation and migration, which regulates the immune cell based autonomous processes [193–196]. Thus, STAT3 blocking has been proposed to be a promising adjuvant for the tumor immunotherapy. Pyrimethamine (PYR), an FDA approved anti-microbial drug, has safe inhibition function for STAT3 [197]. Owing to its immunomodulatory effects, PYR treatment significantly inhibited the metastasis and proliferation of breast cancer cells and remarkably attenuated the density of F4/80^+^ TAMs in breast cancer milieu [30]. Furthermore, PYR-mediated STAT3 inhibition significantly decreased the frequencies of Foxp3^+^ Tregs, while enhanced the frequency of CD8^+^ T cell numbers. The inhibitor against the Y239/240-ShcA phosphorylation site has emerged as a novel therapeutic strategy to inhibit STAT3 activation and increase sensitivity breast cancer cells to immunotherapy. This lead to a direct amelioration of immune suppression in breast cancer and increased tumoricidal properties of immune cells, such as NKs and cytotoxic T cells [15]. In addition, CpG-induced STAT3 activation could counteract by the inhibitor AG490 (STAT3i), which shown the ability to promote the Th1 skewing accumulation by counterbalancing CpG-induced Th2/Tregs and the DC maturation through NF-κB activation [198]. The specific T cell reactivity restores the antitumor immunity in patients with breast cancer, which is affected by release of local cytokine and chemokines like CXC10, CXC9, IFNγ, IL-4 and IL-5 [198]. Furthermore, a natural flavonoid compound, pectolinarigenin, inhibited breast cancer lung metastasis, which simultaneously enhanced the CD8^+^ T cells recruitment and inhibited the STAT3 phosphorylation [158]. The re-activated local immune response is an essential component to sensitize tumors to immunotherapies [199]. Therefore, STAT3 signaling is a most potential therapeutic target for breast cancer systematic immunotherapy.

Previous studies demonstrate that STAT3 activation not merely act as a predictive biomarker for downregulated immune cells response, but also the type of immunomodulator that is strongly associated with programmed death ligand 1 (PD-L1) expression in the TME of breast cancer [15, 29, 200]. The STAT3 translocates to the nucleus where it induces the PD-L1 expression by binding to DNA-response elements in PD-L1 promoter [29, 201]. Disrupting STAT3 cascade could prevent PD-L1 expression [202] and improve the immune surveillance with effector T cells in immune microenvironment [203]. The observation highlighted the therapeutic potentials of targeting STAT3 in TME, especially in breast cancer patients with high PD-L1 expression. Of note, pharmacologic inhibition of STAT3 showed the ability to enhance the efficacy of anti-PD-L1/PD-1 monoclonal antibodies, which was proved effective for patients with metastatic triple negative breast cancer [204]. Ongoing phase I trial (NCT03195699) in advanced-stage breast cancer patients applied the STAT3 SH2-domain binder inhibitor C188-9. Of note, selective inhibition of the SH2-domain by 3-(2,4-dichloro-phenoxymethyl)-5-trichloromethyl-[1,2,4]oxadiazole (ODZ10117) has been reported to significantly inhibit the migration, invasion and distant metastasis of breast cancer [205]. Moreover, a phase II trial combing pSTAT3 inhibitor (napabucasin) with the anti-PD-1 antibody nivolumab in modulating TME is also processing in colorectal cancer (NCT03647839). Therefore, the therapeutic combinations of STAT3 and PD-L1 immune check point inhibition may pave way for the prospective immunotherapy.

## Combing immuno/radio-therapy and STAT3 inhibition in breast cancer

Radiotherapy invokes immune-related responses in cancer by several mechanisms. A growing number of studies have revealed that radiation invokes several systemic biological responses, such as adaptive and innate immune-related activities (T cells, macrophages, Tregs, NKs and CTLs) that affect tumor development [206–211]. In breast cancer, precise radiotherapy favors the local control of treated lesions and evokes the systematic antitumor immunity of tumor-associated antigens (TAAs). For example, stereotactic body radiotherapy (SBRT) further enhanced major histocompatibility complexes (MHCs) expression and immune cells activity in breast cancer [211–214]. While Timaner et al. reported that radiotherapy’s antitumor immune effects can be blunted by mechanisms of immune evasion and immune-suppression, such as radiation-mediated PD-L1 upregulation in the TME of breast cancer [215]. The irradiated tumor cells also increase the secretion of immuno-suppressive factors that promote the infiltration of Tregs, MDSCs, and macrophages [216–218]. These mechanisms potentially limit the anti-tumor effects of radiotherapy. Of note, a better therapeutic strategy, radiotherapy in combination with immune checkpoint inhibition not merely improves the breast cancer therapeutic effect, but also induces abscopal immune responses outside the radiation field [215, 219]. However, the abscopal responses are rarely observed after radiotherapy alone [220]. To data, heterogeneity or complete lack of abscopal response reports in combing immuno/radio-therapy in breast cancer further hindered the ability to define the populations most likely to benefit [220]. Developing predictive biomarkers of treatment response and efficacy in clinically relevant preclinical models is necessary.

Recently, tumor patients receiving standard radiation therapy were benefited from STAT3 inhibition. The STAT3 inhibition in combination with radiation therapy in tumors statistically reduced the radiation-related Tregs and MDSCs accumulation in the TME and further improved the therapeutic effect [221]. In breast cancer, applying the niclosamide to block the STAT3 overcame the radioresistance and significant increase of radiation-induced ROS, which offers an effective alternative approach for improving the breast cancer radiation therapy [222]. Similarly, S3I-201 has been reported to suppress the radiation-induced STAT3 phosphorylation and increase the radiation-induced cell death in breast cancer [223]. STAT3 is multipotent regulator of both tumor cells and immune cells [30, 137, 224]. In addition, there is an evidence that indicates that T cells are indispensable for radiotherapy and STAT3 inhibition synergistic treatment, but the therapeutic efficacy of radiotherapy and STAT3 inhibition cannot preclude the bridge between T cells and myeloid cells [221]. Generally, Tregs depletion alone is not sufficient to orchestrate an anti-tumor immune response, because of deficient TAAs to attract effector T cells infiltration and killing functions [225, 226]. Radiotherapy is well-suited for improving immunotherapy effect through distinct mechanisms, which exposes TAAs, boosts immune chemokines secretion and enhances the diversity of the TCR repertoire of intratumoral T cells [212, 227, 228]. Moreover, it has been reported that in glioma, DCs antigen presentation and T cell effector functions are also enhanced upon combination of STAT3 inhibition and radiotherapy [229]. The survival time and immunological memory were both improved in the synergistic treatment group. Owing to the heterogeneity of breast cancer [230], single immunotherapy strategies are unlikely to achieve uniform, consistent therapeutic responses among all patients. Immune clearance of a tumor is not determined by a solo immune cell population. Therefore, a rational approach to build upon the STAT3 target immunotherapy strategy such as radiation therapy, which might enhance the systematic antitumor immune responses and therapeutic effects in breast cancers.

## Conclusions and future perspectives

Numerous studies support the role of STAT3 in immune cells and dictates the immunomodulatory effects to the TME of breast cancer. Activated STAT3 constitutively suppresses the CD4^+^/CD8^+^ T cells and DCs, and favors the MDSCs, Macrophages and Tregs, implying a key role in breast cancer progression, metastasis and immunity. Here, we provided an outline of STAT3 function in immune cells of breast cancer TME, as well as their cascade gene activation and clinical outcomes. The effects are complex and, in some cases, apparently discrepant. Most likely this could be explained by the differential role of STAT3 in various cell types and its participation in different intracellular pathways. Consistently, immune modulators generation has been described on the one hand as a mechanism of intercellular STAT3 induced TME decay, whereas on the other hand the role of immune cells systematic effect on breast cancer metastasis is influenced by STAT3. The resultant outcome reflects the intricate TME between tumor cells and immune cells with STAT3 aberrant functions. Emerging evidence indicates that targeting STAT3 not merely improves the anti-tumor immunity in TME, but also enhances the immunotherapy effect, therefore rendering STAT3 as a promising therapeutic target. Of note, the radiotherapy in combination with STAT3 target immunotherapy might pave way to further improve types of TME immune cell accumulation, systemic immune response and antitumor therapeutic effect. These avenues provide new opportunity for innovations towards advanced and/or metastasis breast cancer efficient immunotherapy.

In general, most of the immune cells intercellular STAT3 induce immunosuppressive effects and are thus tumor promoted. However, STAT3 has also been partly associated with DCs immune activation and IFN-γ secretion and has anti-tumor properties. Nonetheless, which role of STAT3 determines the outcome is still unclear. Future studies also need to explore pros or cons of STAT3 intervention in regulating immunological environment of breast cancer, especially tumor specific immune cells alteration. In this regard, it will be interesting to determine the diverse abilities of STAT3 in the context of TME regulation and systematic eradication of breast cancer.

## Data Availability

Not applicable.

## Abbreviations

STAT3: Signal transducer and activator of transcription 3
TME: Tumor microenvironment
ER: Estrogen receptor
PR: Progesterone receptor
DCs: Dendritic cells
NKs: Natural killer cells
Tregs: Regulatory T cells
MDSCs: Myeloid-derived suppressor cells
IDO: Indoleamine 2,3-dioxygenase
IRF-8: Interferon regulatory factor–8
ROS: Reactive oxygen species
CSCs: Cancer stem cells
TAMs: Tumor-associated macrophage
HA: Hyaluronan
COX-2: Cyclooxygenase-2
A-FABP: Adipocyte/macrophage fatty acid binding protein
HIF-1α: Hypoxia-inducible factor-1α
TGF-β1: Transforming growth factor β
APCs: Antigen-presenting cells
TDFs: Tumor-derived factors
FLT3L: FMS-related tyrosine kinase 3 ligand
Foxp3: Forkhead box P3
TCR: T-cell receptor
FAO: Fatty acid oxidation
HK2: Hexokinase II
FGFR4: Fibroblast growth factor receptor 4
TAAs: Tumor-associated antigens
SBRT: Stereotactic body radiotherapy
MHCs: Major histocompatibility complexs
